# Optimization of polishing fluid composition for single crystal silicon carbide by ultrasonic assisted chemical-mechanical polishing

**DOI:** 10.1038/s41598-024-77598-x

**Published:** 2024-10-30

**Authors:** Linzheng Ye, Jialong Wu, Xijing Zhu, Yao Liu, Wenlong Li, Shida Chuai, Zexiao Wang

**Affiliations:** 1 School of Mechanical Engineering, North University of China, Taiyuan, 030051 China; 2 Shanxi Key Laboratory of Advanced Manufacturing Technology, North University of China, Taiyuan, 030051 China

**Keywords:** Polishing fluid, Ultrasonic polishing, Orthogonal test, Surface roughness, Material removal rate, Organocatalysis, Process chemistry, Electronic materials, Chemical engineering, Mechanical engineering

## Abstract

Silicon carbide (SiC) is renowned for its exceptional hardness, thermal conductivity, chemical stability, and wear resistance. However, the existing process is difficult to meet the high standards of uniform corrosion in its polishing process and surface roughness and flatness after polishing, new polishing fluids and technique optimization are crucial for development. The study optimized and validated the composition of the polishing fluid used in ultrasonic-assisted chemical-mechanical polishing (UACMP). Abrasives significantly influenced the material removal rate (MRR) and surface roughness (Ra), contributing 67.63% and 56.43%, respectively. Organic bases and pH buffers significantly affected Ra, accounting for 19.66% and 21.44%, respectively. The optimum composition was determined, consisting of triethylamine (3wt%), potassium hydrogen phthalate (1wt%), a composite of silica and alumina abrasive particles (5wt%), and hydrogen peroxide (6wt%), which reduced the Ra from 95 nm to 3 nm. The MRR achieved 25.96 nm/min. In comparison to the 7 nm minimum roughness from the orthogonal test, the optimal scheme’s Ra was reduced by 57.14%, leading to a significant enhancement in overall surface quality. In this paper, a new type of additive is added to prepare the polishing liquid, which provides a new idea for the UACMP of SiC and has a great impact.

## Introduction

Semiconductor materials are essential for modern electronics and optoelectronics, with SiC being a prominent choice due to its exceptional thermal conductivity, electron mobility, chemical stability, and mechanical strength. These attributes endow SiC with significant potential for use in high-frequency, high-power, and high-temperature electronic devices^1–8^. However, the broad application of SiC is constrained by advancements in its surface processing technology. Specifically, controlling surface flatness and microscale quality is critical for SiC materials. The SiC surface must be ultra-smooth, defect-free, and undamaged. Nonetheless, the high hardness, brittleness, and chemical inertness of single-crystal SiC pose significant challenges to efficient ultra-precision surface processing^9–12^. Thus, overcoming these surface processing challenges in SiC is a key area of current research.

UACMP is currently an effective technology for reducing the surface roughness of SiC, and ultrasonic and polishing fluid formulations are essential for efficient and low-damage polishing in CMP. Cui et al.^13^ added ultrasonic vibration to the polishing of SiCp/Al ceramics, showing that the introduction of ultrasonic can greatly improve the material removal rate and maximum removal depth. Zhou et al.^14^ added ultrasonic to the polishing of sapphire, and the research results showed that the damage layer on the pressed surface of sapphire was much thinner and more uniform after the addition of ultrasonic polishing.Recent research on SiC UACMP polishing liquids has concentrated on abrasive selection, additive chemistry, concentration ratios, and considerations for environmental sustainability and safety. Commonly used abrasives in SiC polishing include alumina (Al_2_O_3_), cerium oxide (CeO_2_), and silicon dioxide (SiO_2_)^15–19^. These abrasives remove SiC surface material through both chemical and physical interactions. Song et al.^20^ investigated how SiO_2_ solid content affects the removal rate of N-type monocrystalline silicon during chemical-mechanical polishing, noting an increase in the rate with higher SiO_2_ content. Song et al.^21^ examined the polishing performance of CeO_2_ in alkaline solutions on silicon wafers, observing a positive correlation between CeO_2_ concentration and material removal rate. Yuan et al.^22^ developed ethylenediaminetetraacetic acid grafted cerium oxide (CeO_2_-EDTA) composite abrasives, which increased the material removal rate by approximately 40% and enhanced surface quality compared to pure CeO_2_. Xu et al.^23^ created a series of Ce_1 − 2x_Y_x_Pr_x_O_2_ abrasives using the molten salt method, significantly enhancing both the material removal rate and surface quality of polished quartz glass, resulting in a smooth, defect-free, and low-roughness surface.

The efficacy of SiC UACMP can be augmented by incorporating various chemical additives into the polishing fluid. For instance, incorporating oxidants like H_2_O_2_ facilitates chemical reactions on the SiC surface, and the inclusion of acidic or alkaline agents modulates the polishing fluid’s pH, thereby influencing UACMP efficiency and quality^24–29^. Lu et al.^27^ enhanced the surface quality of monocrystalline silicon through chemical-mechanical polishing by incorporating isopropyl alcohol. As the IPA concentration rose from 0.1 mol/L to 2.5 mol/L, both surface quality and the material removal rate (MRR) improved. At 2.5 mol/L, the IPA concentration resulted in a surface roughness more than 58.3% lower than that achieved without IPA. Xu et al.^30^ introduced a pH buffer (sodium tetraborate) into the polishing fluid, determining the optimal formulation through experimental trials. The organic base had a mass fraction of 1.0%, while the pH buffer (sodium tetraborate) constituted 1.1%, with a pH stabilizer added to regulate the pH. The polishing removal rate, fluid longevity, and surface roughness saw significant enhancements. Zou et al.^31^ investigated the impact of KOH, aminomethylpropanol (AMP), and arginine (ARG) as pH regulators on the CMP properties of sapphire with C-side, A-side, and R-side orientations. ARG was found to be the most effective, enhancing both the material removal rate and surface quality. Dong et al.^32^ compared the effects of the inorganic pH regulator KOH, the organic pH regulator diethanolamine (DEA), and 2-amino-2-methyl-1-propanol (AMP) on Cu membrane CMP and polishing fluid properties. AMP, when used as a pH regulator, achieved a Cu/Ru removal rate selectivity (RRS) of 598 and served multifunctional roles as a complexing agent, dispersant, and surfactant. Yan et al.^33^ incorporated 2 wt% NaHCO_3_ as a pH buffer into the polishing fluid for GaSb wafer CMP. The polished surfaces exhibited high quality, free of scratches, surpassing the quality of the most advanced GaSb wafers commercially available. Xu et al.^34^ investigated the effects of iron catalysts, including Fe, FeO, Fe_2_O_3_, and Fe_3_O_4_, produced via the Fenton reaction, on the polishing fluid’s performance in chemical-mechanical polishing (CMP) of single-crystal SiC. The findings indicate that utilizing Fe_3_O_4_ as a catalyst facilitates a pronounced chemical reaction on the SiC surface, resulting in the formation of a soft SiO_2_ oxide layer that can be easily removed. Liang et al.^35^ polished the carbon and silicon surfaces of 4 H-SiC using a potent oxidizing CMP liquid with KMnO_4_, discovering that an optimal concentration of KMnO_4_ significantly enhanced both the CMP removal rate and the surface quality of the 4 H-SiC substrate. Wang et al.^36^ examined the impact of varying ethylenediamine (EDA) concentrations on silicon polishing rates, revealing that an increase in EDA concentration led to a proportional rise in the polishing rate, peaking at a 74.5% increase at a 5% mass fraction. A.M. Hamed et al.^37^ investigated the influence of specific amines on steel electropolishing, finding that varying concentrations of these organic amines could suppress the electrolytic polishing process, with inhibition efficiencies ranked as: triethanol amine > diethanol amine > triethyl amine > diethyl amine > methyl amine. Wang et al.^38^ explored the corrosion inhibition capabilities of nitrogen-containing organic compounds in alkaline copper polishing solutions, discovering that the quantity of heteroatoms and the substitution pattern of the amino group enhanced the compounds’ corrosion inhibition efficiency. Huo et al.^39^ incorporated N, N’-bis (3-aminopropyl) ethylenediamine (TAD) into the CMP process for copper, mitigating uneven copper corrosion and achieving high material removal rates along with optimal surface finish. With a growing emphasis on environmental sustainability, researchers are increasingly seeking greener and safer polish formulations, focusing on minimizing or eliminating the use of toxic chemicals.

Orthogonal experimental design, a method for multi-factor and multi-level experimentation, strategically selects test points that are “uniformly dispersed and neatly comparable” to minimize testing while acquiring ample information. Its efficiency, speed, and cost-effectiveness have led to widespread adoption across various research domains. Yin et al.^40^ performed an orthogonal test to analyze factors impacting the characteristics of lead-zinc tailings cementation material; Chen et al.^41^ applied the same method to study the low-temperature properties of butadiene-modified asphalt; Chen et al.^42^ enhanced the ultrasonic polishing of silicon carbide using a Taguchi method and grey relational analysis for multi-objective optimization. Accordingly, this study’s optimization of the polishing liquid composition employs the orthogonal test methodology.

In this study, hydrogen peroxide (H_2_O_2_) serves as the oxidant, with ethylenediamine, triethylamine, and tetramethylammonium hydroxide as organic bases, and sodium tetraborate, mixed phosphate, and potassium hydrogen phthalate as pH buffers. Abrasives include single SiO_2_, SiO_2_/Al2O_3_, and SiO_2_/CeO_2_ composites. Orthogonal experiments were conducted, targeting surface roughness and material removal rate for optimization, with the optimal polishing liquid components identified through range analysis. The impact of the organic base, pH buffer, and abrasive particles on the optimization criteria was evaluated using variance analysis, leading to the formulation of a novel UACMP polishing liquid for single-crystal silicon carbide. Ultimately, the UACMP experiment was executed using the optimal polishing fluid mixture, followed by a verification analysis of this optimal combination.

## Experiment

**Fig. 1:**
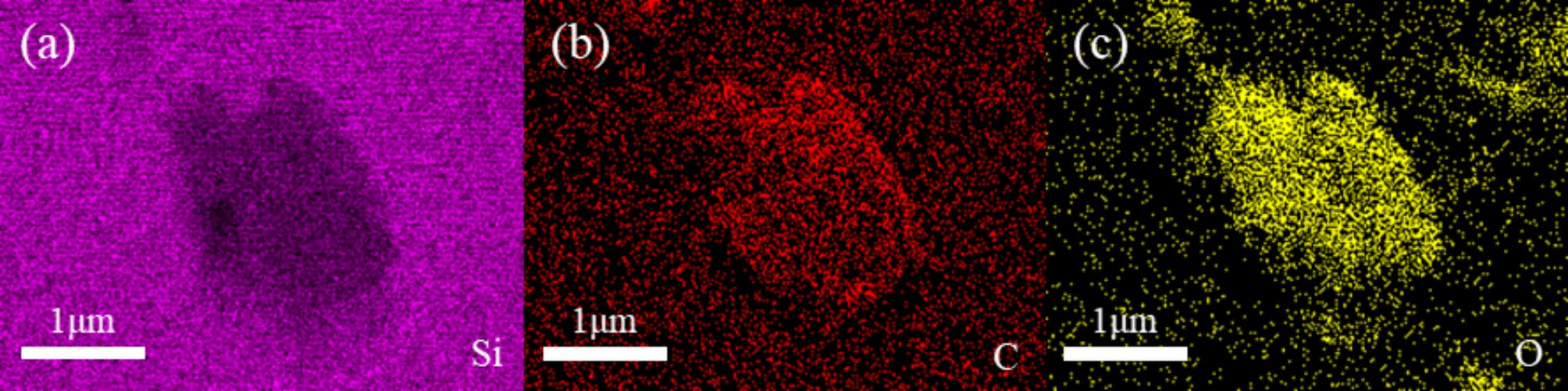
SEM image of single crystal silicon carbide.

The dimensions of the polished silicon carbide are 10 mm × 10 mm × 1 mm, with a chemical composition of Si 55.51%, C 33.12%, and O 11.37%. Figure 1 presents the SEM image of the polished single-crystal silicon carbide.

**Fig. 2:**
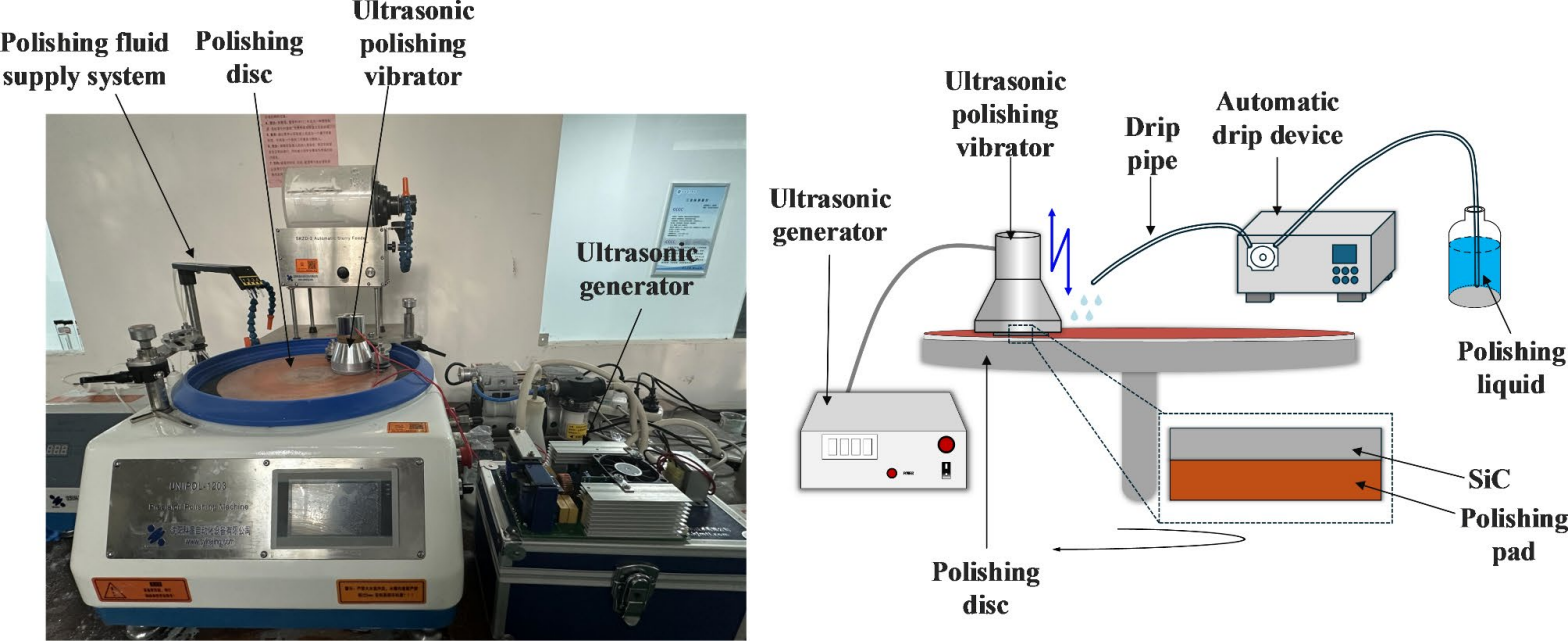
Schematic diagram of polishing device and polishing system.

Silicon carbide samples were ultrasonically cleaned before and after the polishing experiments. In UACMP, the polishing solution contained organic bases, pH buffers, abrasive grains, and oxidizing agents. The organic base had a mass fraction of 3%; referencing optimal ratios from existing studies, the pH buffer’s mass fraction was set at 1%^30^, abrasive grains at 5% with a particle size of 50 nm, and the oxidizing agent at 6%. Ethylenediamine (EDA), triethylamine (TEA), and tetramethylammonium hydroxide (TMAH) were chosen as organic bases; sodium tetraborate (Na_2_B_4_O_7_), mixed phosphates, and potassium hydrogen phthalate (KHP) as pH buffers; silicon dioxide (SiO_2_), a mixture of silica and alumina (Al_2_O_3_), and silica combined with cerium oxide (CeO_2_) as abrasives; and hydrogen peroxide (H_2_O_2_) as the oxidizing agent. The polishing pads, constructed from non-fluted polyurethane, were utilized with a UNIPOL-1203 chemical-mechanical grinding and polishing machine by Shenyang Kejing Automation Equipment Co. The polishing pad rotated at 200 rpm, exerted a pressure of 11.76 N, supplied the polishing solution at a rate of 5 ml/min, operated for a duration of 2 h, with an ultrasonic frequency of 20 kHz and an amplitude of approximately 2 μm. The schematic diagram of the specific experimental device and polishing system is depicted in Fig. 2. The ultrasonic generator drives the ultrasonic vibrator to produce longitudinal vibration, the silicon carbide sheet is glued to the bottom of the ultrasonic vibrator with paraffin wax and pressed on the polishing pad, which is suctioned to the polishing disk through the magnetic suction piece and rotates with the polishing disk in accordance with the set rotational speed, and at the same time, an automatic dripping device extracts the polishing liquid in the bottle and drips it on the polishing pad in accordance with the set speed.

Orthogonal testing efficiently extracts comprehensive experimental insights with a reduced number of trials, utilizing three levels for each of the three factors, with the L_9_ (3^4^) design selected for the orthogonal test, and includes a blank column designated for the error term. The test criteria are the material removal rate and surface roughness, the material removal rate post-UACMP testing is determined using Eq. (1), and the post-polishing surface roughness of SiC is measured using a confocal microscope, with measurements taken over a 128 μm × 128 μm area at five distinct locations on the polished surface. The test factors and levels are detailed in Table 1, and the test protocol is presented in Table 2. A total of 9 test groups are executed.

1 $$MMR=\frac{{\Delta m \cdot 10_{{}}^{4}}}{{s \cdot \rho \cdot t}}$$

where *Δm* is the mass difference of SiC before and after ultrasound-assisted chemical-mechanical polishing, *s* is the surface area of silicon carbide, and *ρ* is the density of SiC 3.21 g/cm^3^, *t* is the polishing time.

**Table 1:** Test factors and levels.

Level	Considerations		
	Organic base	pH buffer	Abrasive particle
1	Ethylenediamine	Sodium tetraborate	SiO_2_
2	Triethylamine	Mixed phosphate	SiO_2_/Al_2_O_3_
3	Tetramethylammonium hydroxide	Potassium hydrogen phthalate	SiO_2_/CeO_2_

## Results and discussion

### Orthogonal test results

**Fig. 3:**
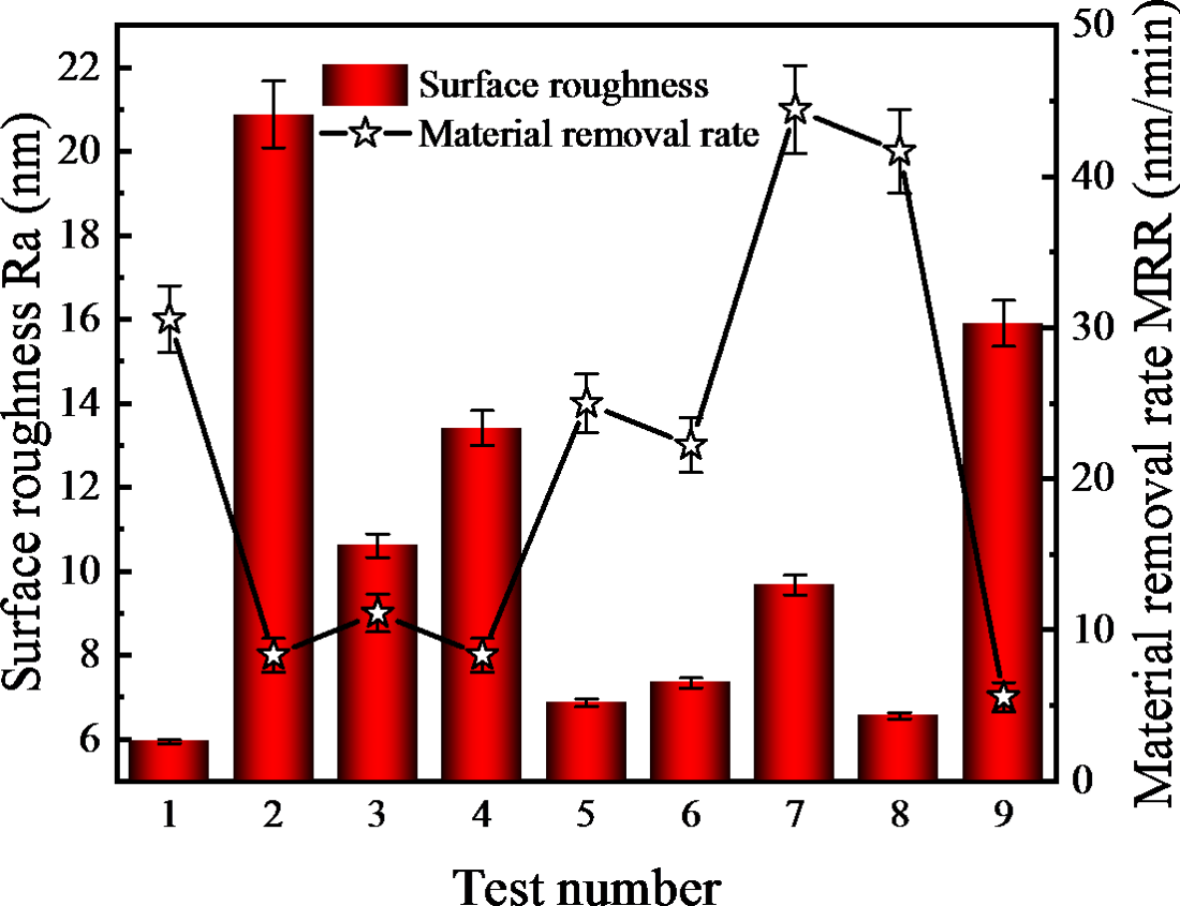
Material removal rate and surface roughness results of orthogonal test.

**Fig. 4:**
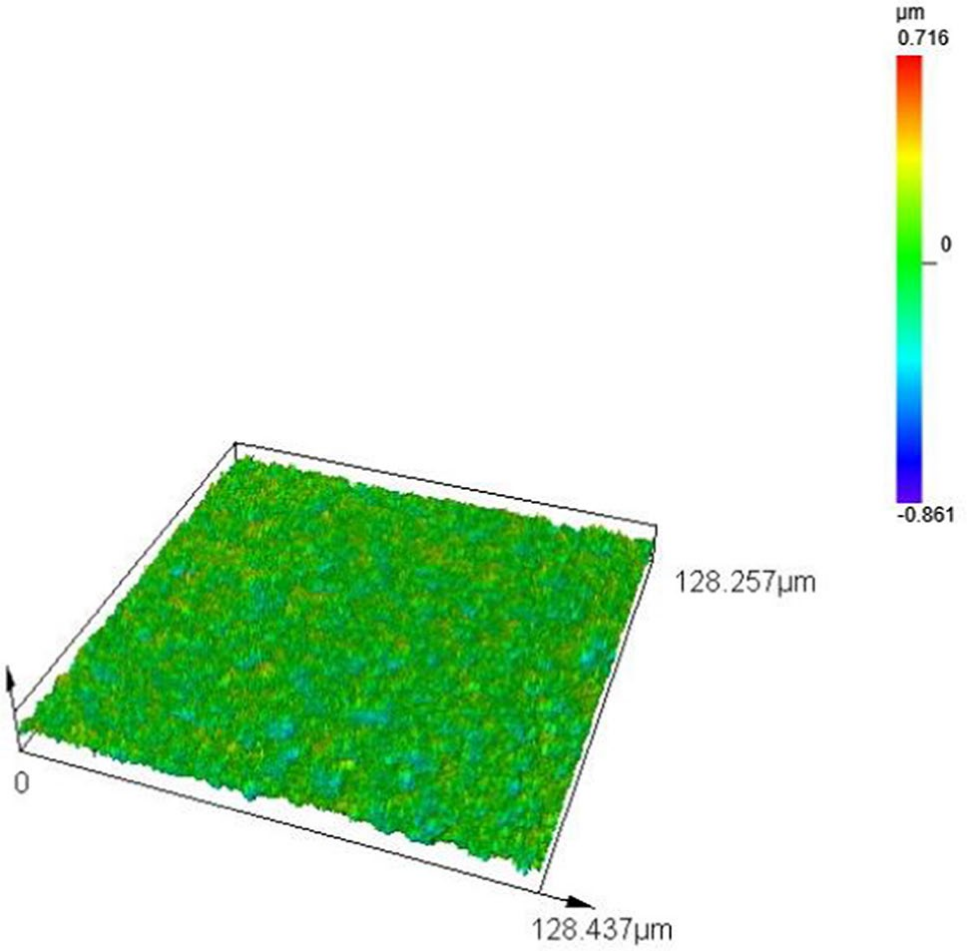
3D topography of the original surface before polishing.

The outcomes of laser confocal microscopy inspections, which detail the material removal rate and surface roughness, are presented in Table 2 and depicted in Fig. 3. Figure 3 demonstrates substantial variation in the material removal rate and surface roughness following UACMP with the nine polishing fluids, to document the surface morphology alterations pre- and post-polishing, the 3D surface topography of the samples was examined using confocal microscopy, the pre-polishing 3D surface topography is depicted in Fig. 4, with an average roughness (Ra) of approximately 95 nm, the initial surface features numerous bumps of diverse elevations.

**Table 2:** Test scheme and results.

Test number	Considerations			index	
	Organic base	pH buffer	Abrasive particle	Material removal rate MRR/(nm/min)	Surface roughness Ra/(nm)
1	Ethylenediamine	Sodium tetraborate	SiO_2_	2.60	16
2	Ethylenediamine	Mixed phosphate	SiO_2_/Al_2_O_3_	44.13	8
3	Ethylenediamine	Potassium hydrogen phthalate	SiO_2_/CeO_2_	15.58	9
4	Triethylamine	Sodium tetraborate	SiO_2_/Al_2_O_3_	23.36	8
5	Triethylamine	Mixed phosphate	SiO_2_/CeO_2_	5.19	14
6	Triethylamine	Potassium hydrogen phthalate	SiO_2_	6.49	13
7	Tetramethylammonium hydroxide	Sodium tetraborate	SiO_2_/CeO_2_	12.98	21
8	Tetramethylammonium hydroxide	Mixed phosphate	SiO_2_	4.33	20
9	Tetramethylammonium hydroxide	Potassium hydrogen phthalate	SiO_2_/Al_2_O_3_	30.29	7

**Fig. 5:**
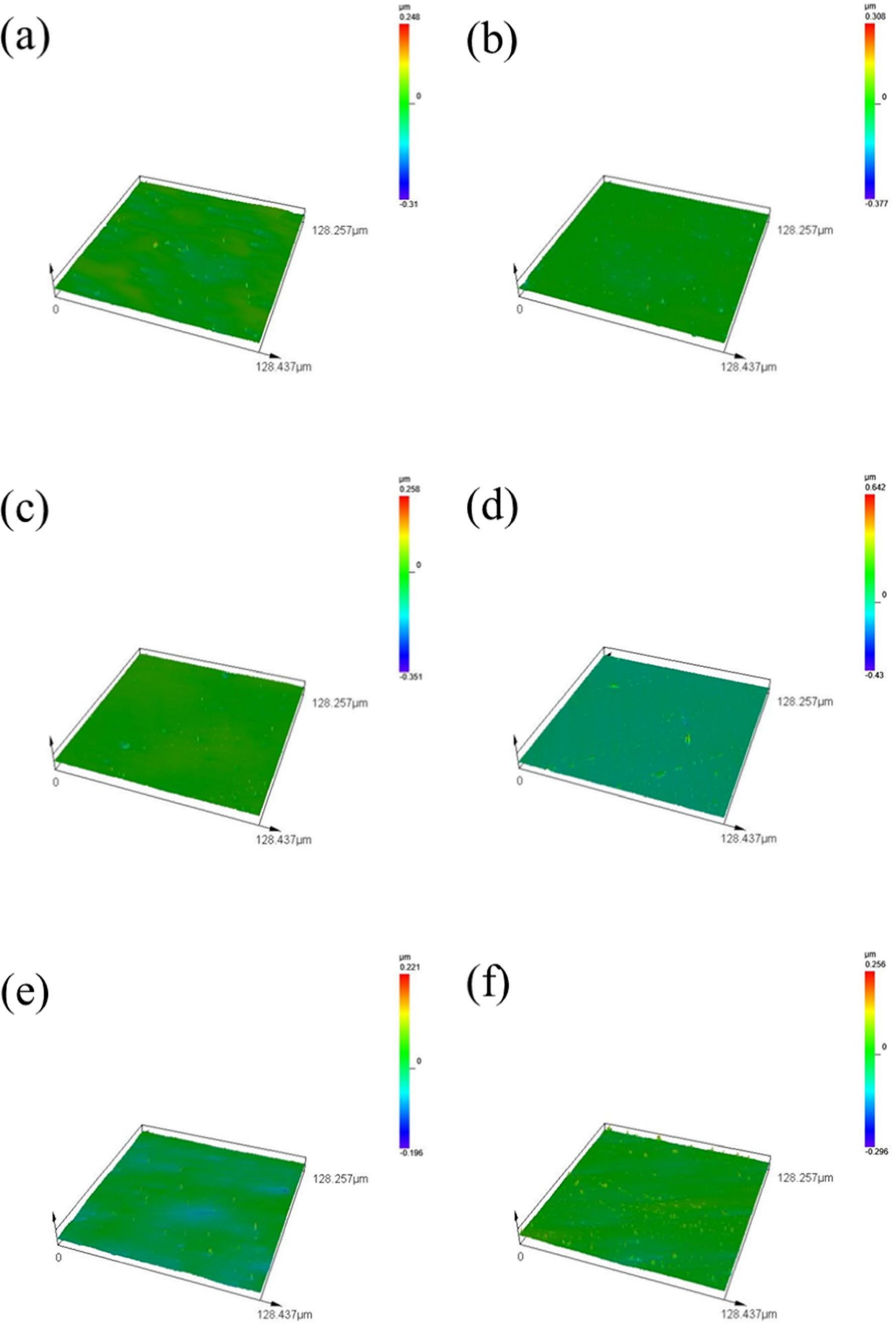

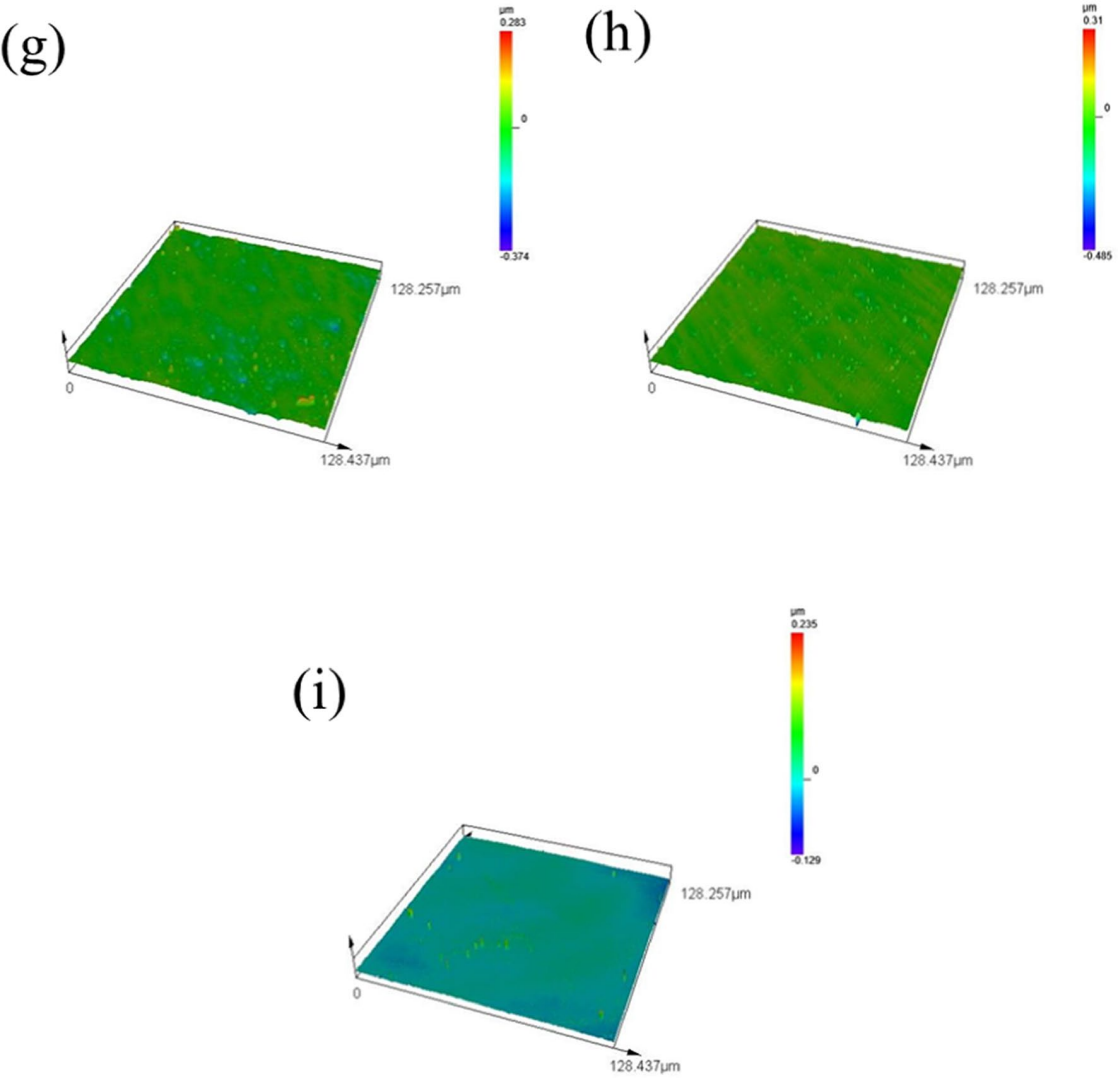
3D surface topography after ultrasonic assisted chemical-mechanical polishing.

Figure 5a to i represent the nine groups of orthogonal test experiments, each with an observation area of 128.257 μm × 128.437 μm. After 120 min of UACMP, Ra dropped to the range of 5–25 nm, and the surface bulges were significantly reduced. Nonetheless, Fig. 5f, g, and h show numerous bumps of varying sizes on the SiC surface post-polishing with the 6th, 7th, and 8th fluids, suggesting suboptimal interactions among the organic base, pH buffer, abrasives, and oxidizers, and that the material selections and concentrations did not meet the desired outcomes. The organic base and pH buffer may not provide adequate anti-corrosion properties, leading to excessive SiC surface corrosion.

If the corrosion layer isn’t promptly polished and removed, the chemical reaction rate could exceed the material removal rate. The fewest remaining surface bulges are observed in group 9, as depicted in Fig. 5i, indicating effective protection of the SiC surface from over-etching by the organic base and pH buffer. These agents maintain the polishing fluid’s pH stable, ensuring a balanced and steady chemical reaction and polishing rate.

### Analysis of range variance

The order of influence of the factors on the indexes was investigated by polar analysis, and it was found that the order of influence of the factors was abrasive grain C, organic alkali A, and pH buffer B by calculating the k-value and the R-value, no matter whether it was for the material removal rate or for the surface roughness.

Upon comparing the k-values in Table 3, the optimal combination for maximizing material removal rate was determined to be A_1_B_2_C_2_. This configuration corresponds to the use of ethylenediamine as the organic base, mixed phosphate as the pH buffer, and a mixture of silica and alumina as abrasive particles, yielding the highest removal rate. Comparison of k-values in Table 4 identified A_2_B_3_C_2_ as the optimal combination for minimizing surface roughness. This combination involved triethylamine as the organic base, potassium hydrogen phthalate as the pH buffer, and a mixture of silica and alumina as abrasive particles, resulting in the lowest roughness values. The optimal surface roughness combination, A_2_B_3_C_2_, was not among the nine tested configurations, underscoring the benefit of orthogonal testing in identifying effective factor combinations that may not be immediately apparent.

**Table 3:** Material removal rate range analysis.

Test index	Test number	Organic base A	pH buffer B	Abrasive Particle C
Material removal rate MRR (nm/min)	K_1_	62.31	38.94	13.42
	K_2_	35.04	53.65	97.78
	K_3_	47.60	52.36	33.75
	k_1_	20.77	12.98	4.47
	k_2_	11.68	17.88	32.59
	k_3_	15.87	17.45	11.25
	Range R	9.09	4.9	28.12
	Primary and secondary level	CAB		
	Optimal level	A_1_	B_2_	C_2_
	Optimal combination	A_1_B_2_C_2_		

**Table 4:** Surface roughness range analysis.

Test index	Test number	Organic base A	pH buffer B	Abrasive Particle C
Surface roughness Ra (nm)	K_1_	57	45	49
	K_2_	35	42	23
	K_3_	48	29	44
	k_1_	19.00	15.00	16.33
	k_2_	11.67	14.00	7.67
	k_3_	16.00	9.67	14.67
	Range R	7.33	5.33	8.66
	Primary and secondary level	CAB		
	Optimal level	A_2_	B_3_	C_2_
	Optimal combination	A_2_B_3_C_2_		

The significance of the impact of various factors on the performance metrics can be examined via variance analysis, as demonstrated in Tables 5 and 6.

**Table 5:** Material removal rate variance analysis.

Experimental index	Source of variance	Deviation sum of squares	Degree of freedom	F-ratio	F critical value	S%	Significance
Material removal rate MRR (nm/min)	A	124.199	2	1.501	6.940	7.64%	
	B	44.238	2	0.535	6.940	2.72%	
	C	1292.195	2	15.614	6.940	79.46%	*
	error	165.52	4			10.18%	

**Table 6:** Variance analysis of surface roughness.

Experimental index	Source of variance	Deviation sum of squares	Degree of freedom	F-ratio	F critical value	S%	Significance
Surface roughness Ra (nm)	A	44.222	2	7.959	19.000	19.66%	
	B	48.222	2	8.679	19.000	21.44%	
	C	126.889	2	22.838	19.000	56.43%	*
	error	5.56	2			2.47%	

*α = 0.05 is significant.

**Fig. 6:**
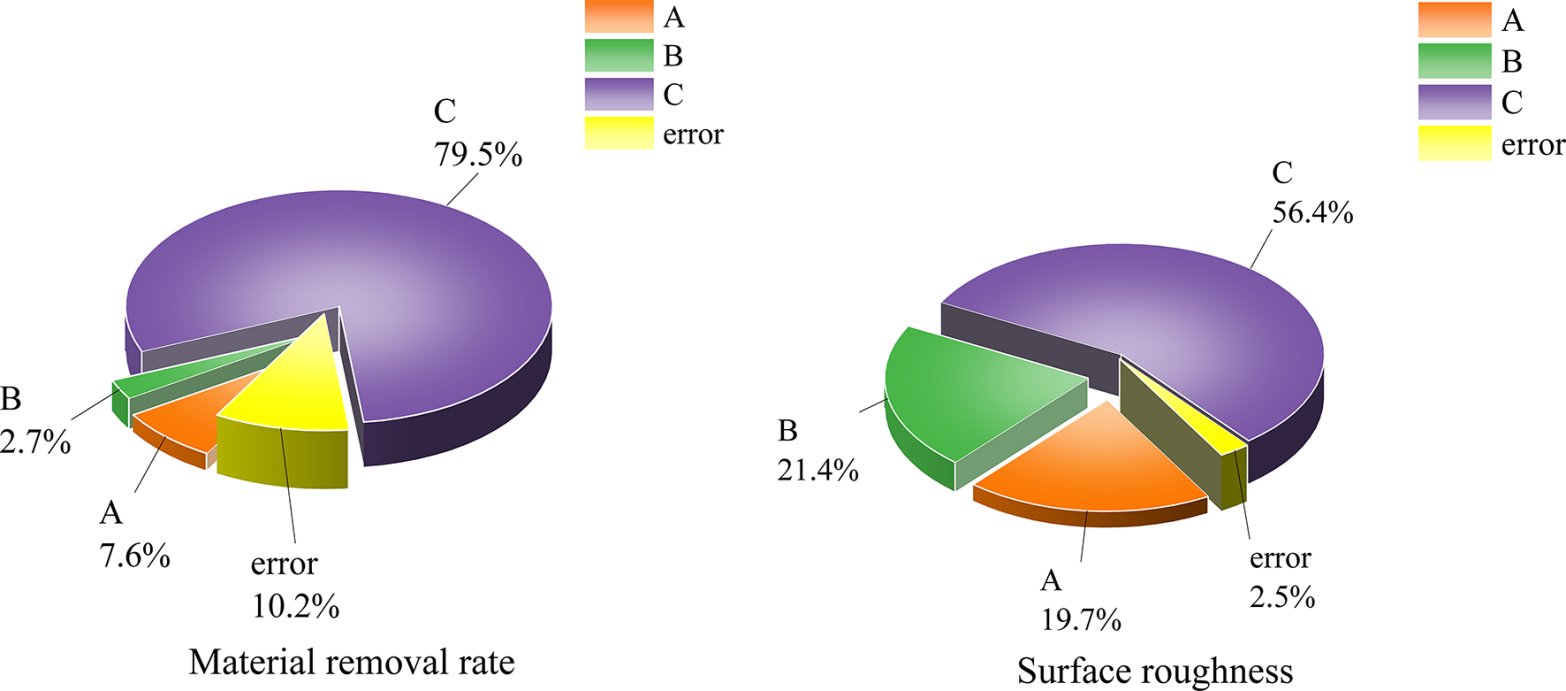
Contribution of each factor to material removal rate and surface roughness.

The contribution of each factor to the material removal rate and surface roughness is shown in Fig. 6. Variance analysis in Tables 5 and 6 indicates that, with 95% confidence, factor C significantly affects both the material removal rate and surface roughness, contributing 67.63% and 56.43% respectively.Factors A and B significantly affect surface roughness, with contributions of 19.66% and 21.44% respectively, leading to the determination of the optimal combination as A_2_B_3_C_2_.

**Fig. 7:**
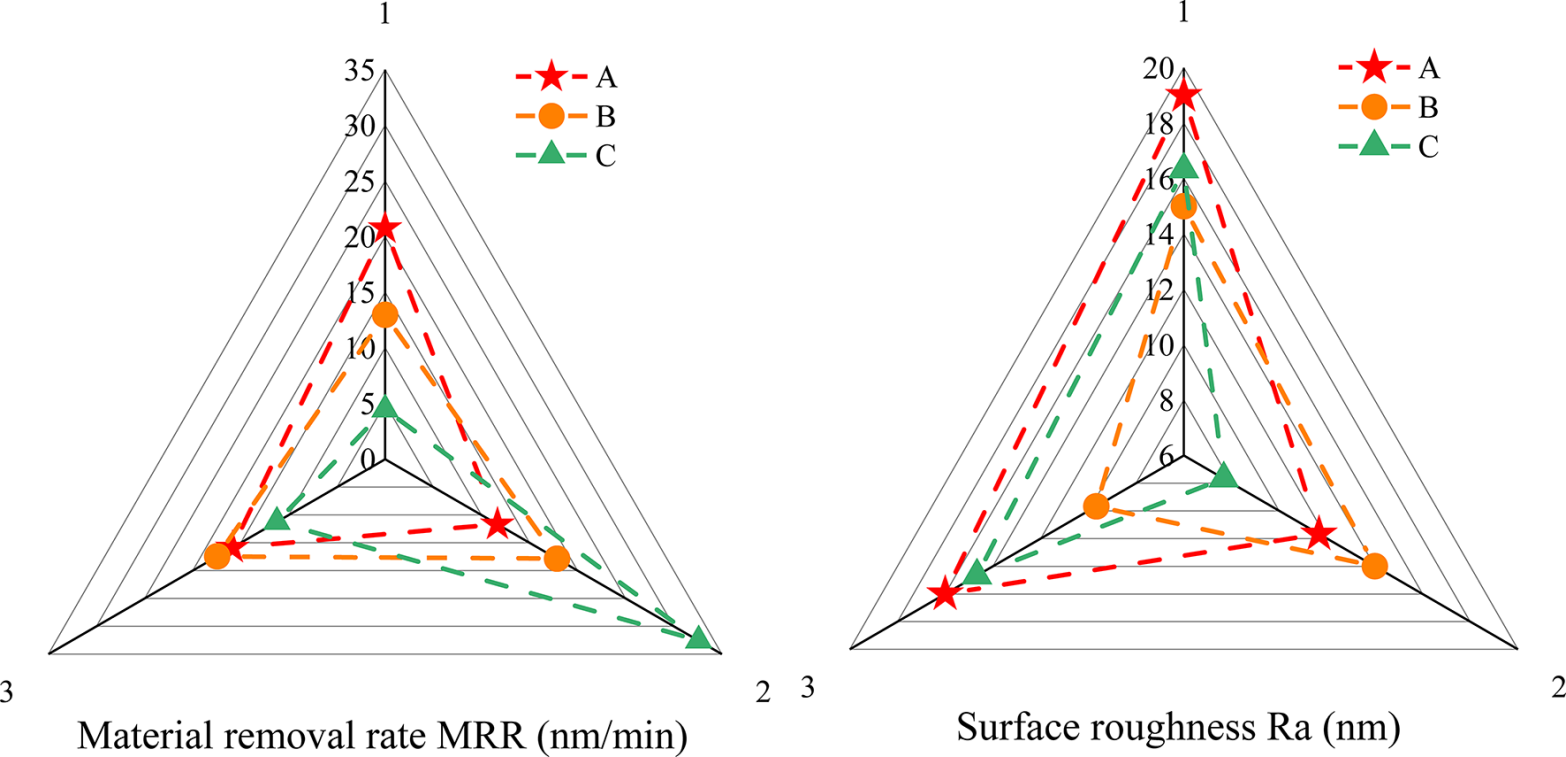
Material removal rate and surface roughness at different levels of single factor. Note that A, B, and C represent organic base, PH buffer, and abrasive particles respectively. 1, 2, and 3 represent the three levels of each factor.

Figure 7 details the distribution of material removal rates and surface roughness across varying levels of individual factors. Figure 7 indicates that the highest material removal rate is achieved with EDA as the organic base, possibly due to its ability to alter the silicon surface’s charge distribution, enhancing chemical reactivity^36^. This suggests that EDA more effectively facilitates the chemical reaction between the SiC surface and the polishing liquid. The lowest surface roughness is observed with TEA, potentially because its higher corrosion inhibition efficiency compared to EDA better protects the SiC surface from excessive etching^37^. Among buffering agents, mixed phosphates yielded the highest material removal rate, while potassium hydrogen phthalate (KHP) resulted in the lowest surface roughness. Mixing silica and alumina achieves the highest material removal rate and the lowest surface roughness. Different types of abrasives have different physical and chemical properties, so the mixed use can give full play to the advantages of each abrasive, thereby improving the polishing effect.

### Experimental verification of optimal combination

The optimal formulation, A_2_B_3_C_2_, comprising triethylamine, potassium hydrogen phthalate, silicon dioxide, and alumina, as determined by the three-factor, three-level orthogonal test, was used to prepare the polishing fluid for the UACMP silicon carbide experiment. Polyester pads were selected as the polishing interface. The polishing disc rotated at a speed of 200 rpm, exerted a pressure of 11.76 N, and the polishing fluid was supplied at a rate of 5 mL/min. The experiment was conducted for a duration of 2 h with an ultrasonic frequency of 20 kHz and an amplitude of approximately 2 μm.

**Fig. 8:**
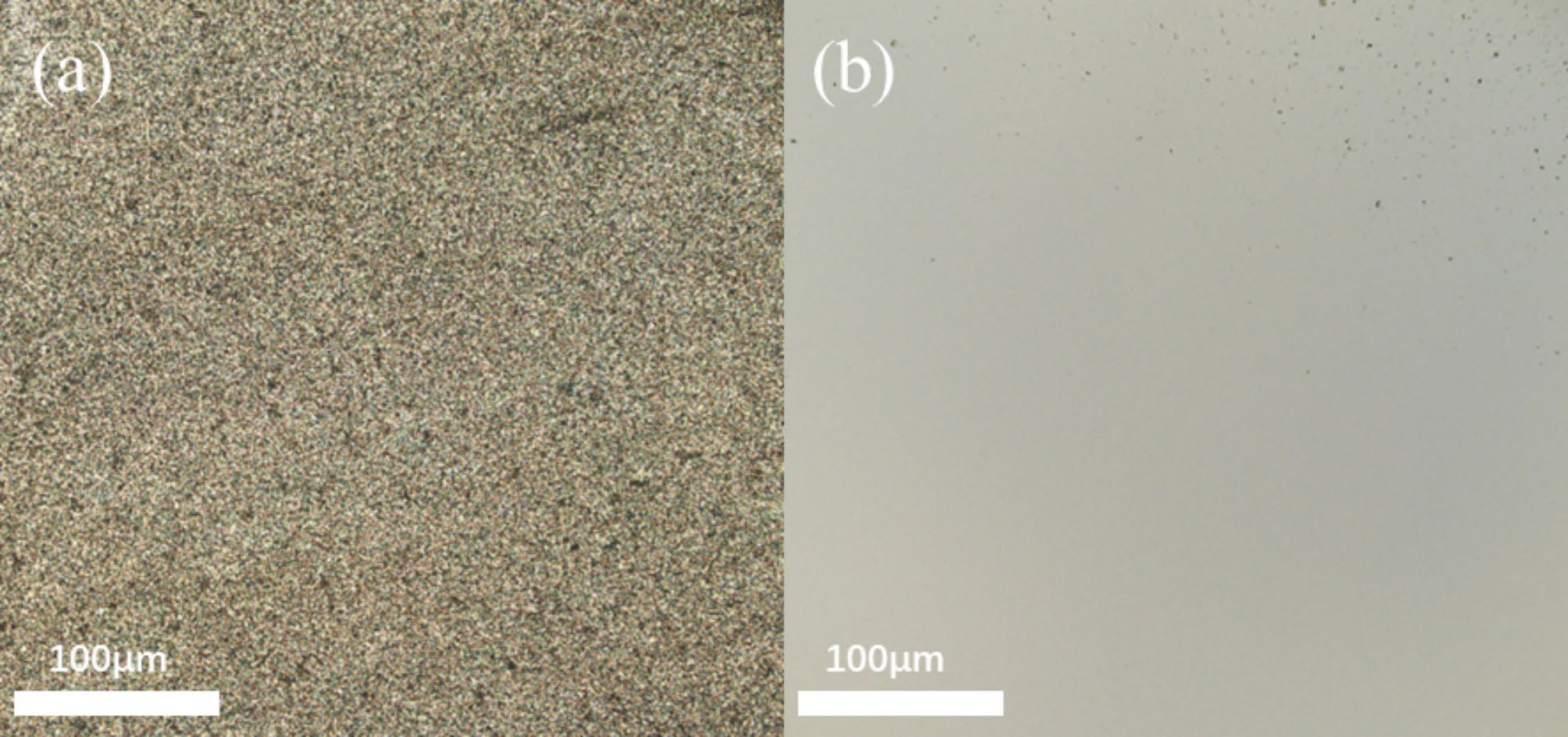
Surface topography of silicon carbide before (**a**) and after (**b**) optimization experimental polishing.

**Fig. 9:**
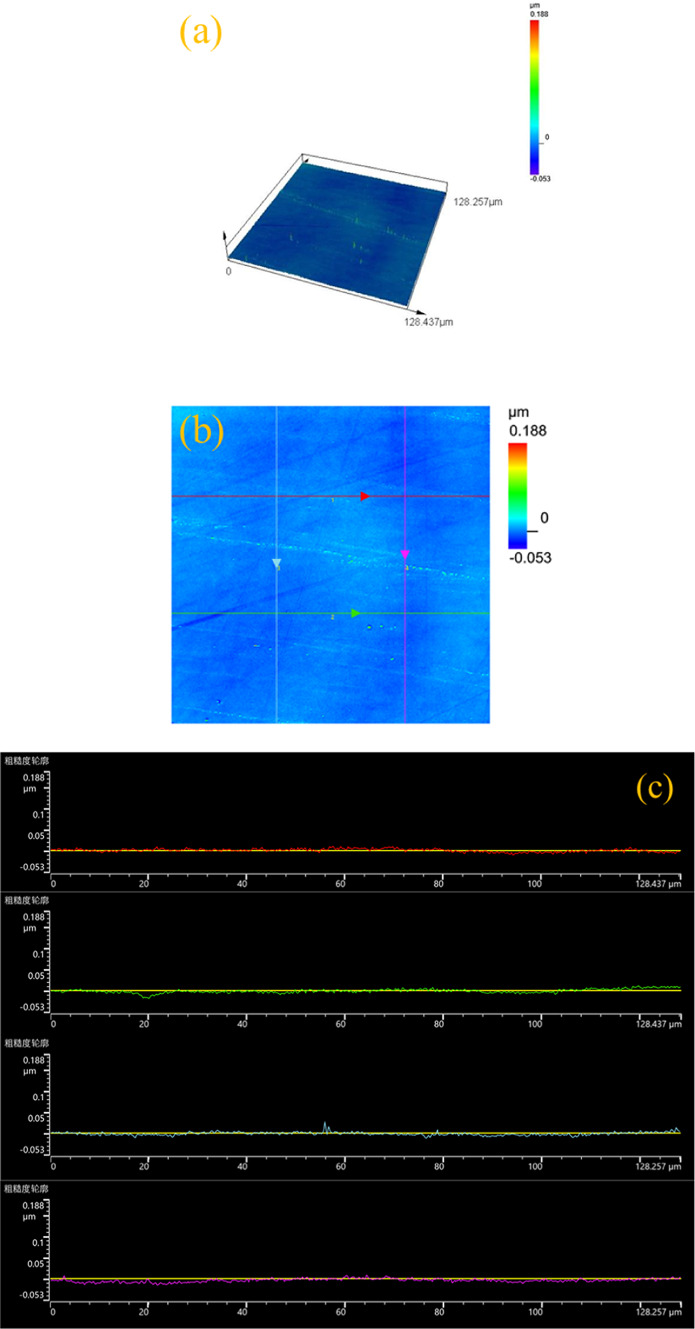
3D topography (**a**) and surface line profile (**b**) and (**c**) of polished silicon carbide surface of the optimal group.

After UACMP, the surface roughness measured using a laser confocal microscope was 3 nm, and the difference in mass before and after was measured using a precision electronic scale, and the material removal rate was calculated to be 25.96 nm/min. Compared to the 7 nm surface roughness obtained from the orthogonal experiments, the optimized experiment achieved a 57.14% reduction. The surface morphology of SiC, both pre- and post-optimized polishing, is depicted in Fig. 8. Figure 8a shows a large number of tiny bumps and some inter-crystalline pores, after polishing (Fig. 8b) tiny bumps and inter-crystalline pores are significantly reduced, and the polished silicon carbide surface is bright white compared with the surface of the silicon carbide before polishing, and the surface finish is significantly improved, but due to the presence of a small number of tiny bumps and inter-crystalline pores, there are still defects on the surface of the polished silicon carbide. Figure 9a presents the 3D topography of the SiC surface post-polishing using the optimal polishing liquid composition. In comparison to Figs. 4 and 5, the optimal group’s post-polishing surface quality shows marked improvement, being generally smooth and flat, albeit with a few remaining small bumps.

**Fig. 10:**
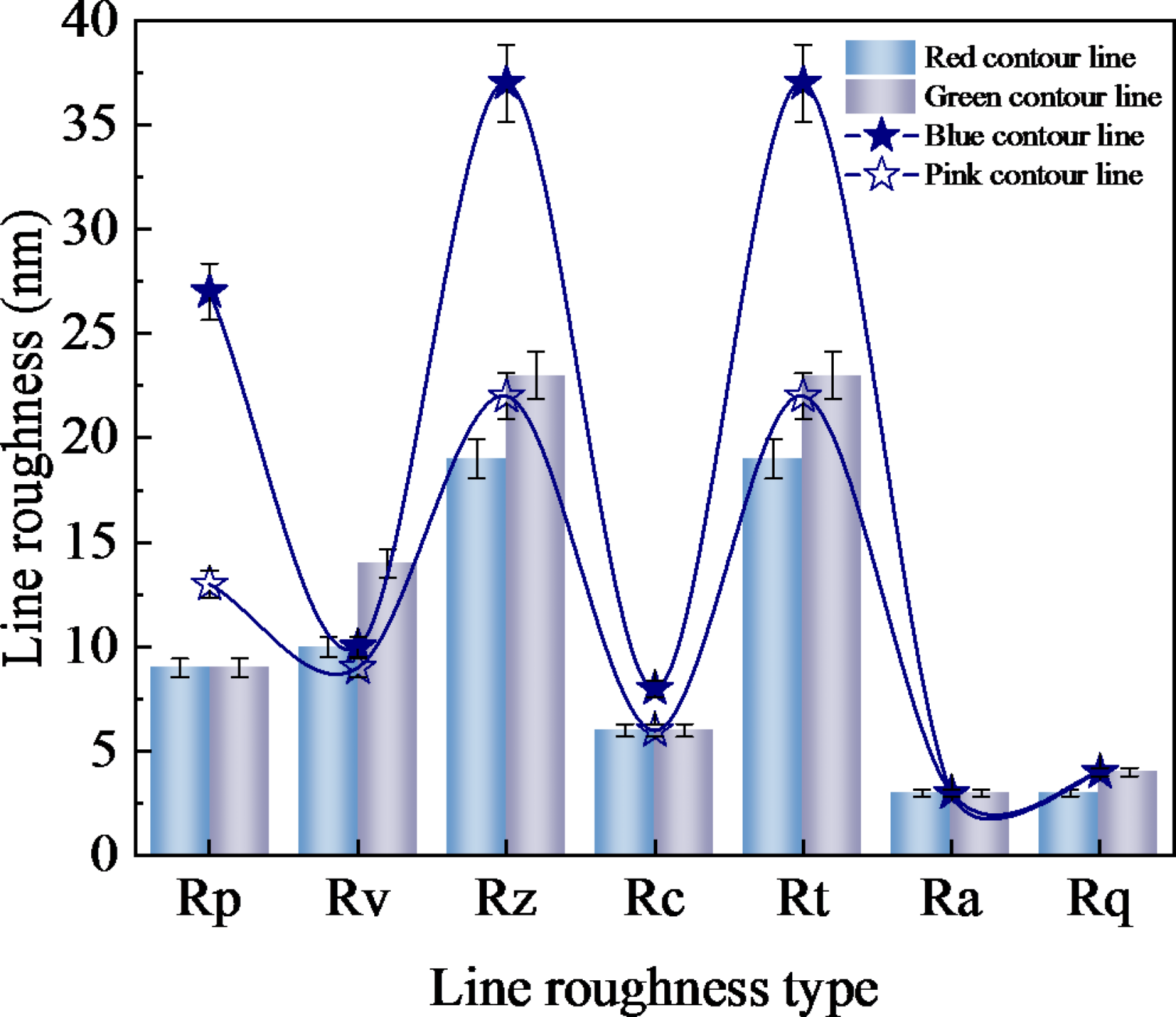
Line roughness, where Rp is the maximum peak height, Rv is the maximum valley bottom depth, Rz is the average of the 10 maximum peaks and the maximum valley bottom, Rc is the maximum peak peak value, Rt is the total height of the 10 maximum peaks and the maximum valley bottom, Ra is the average roughness, Rq is the root-mean-square roughness.

Figure 9b, c display the surface line profile of SiC following polishing with the optimal composition polishing liquid. The line roughness is measured by taking the red, green, blue and pink lines in 9b, and the line profile corresponds to the red, green, blue and pink lines in 9c. The contour line fluctuation after polishing in the optimum group was very small, once again verifying the improvement of surface roughness and surface finish. Figure 10 shows the various types of line roughness of the contour line. Figure 10 reveals that the average roughness (Ra) of the four lines is consistent at 3 nm, confirming the smooth and uniform surface quality of the optimal group.

## Conclusion

UACMP is an efficient and widely used technique for polishing silicon carbide. An orthogonal test was employed to ascertain the relative impacts of the organic base, pH buffer, and abrasive on both the surface roughness and material removal rate of silicon carbide. The primary outcomes are summarized below:

1. A range variance analysis was conducted to assess the impact of the chosen materials on the polishing attributes. The degree of influence on the material removal rate and surface roughness follows this order: abrasive particles, organic base, and pH buffer. The combination of ethylenediamine, mixed phosphate, silica, and alumina exerts the most significant influence on the material removal rate. Conversely, the combination of triethylamine, potassium hydrogen phthalate, silica, and alumina most profoundly affects surface roughness. After evaluating the contributions of various factors to both surface roughness and material removal rate, the optimal combination was identified as triethylamine, potassium hydrogen phthalate, silica, and alumina.
2. Ultrasonic-assisted chemical-mechanical polishing (UACMP) was conducted using the optimal combination of components. After polishing, the surface roughness of SiC was 3 nm, and the material removal rate was 25.96 nm/min. Compared to the nine sets of orthogonal experiments, the surface roughness was reduced by 57.14%, the surface micro bumps and intergranular pores were significantly reduced, and compared with the surface of silicon carbide before polishing was bright white, the surface finish is significantly improved.
3. The line profile and roughness of its four lines were observed using confocal microscope, the line profile fluctuation was very small and the average roughness was 3 nm for all of them, which again verified the improvement of the surface roughness and finish of the silicon carbide polished under the optimal combination of the composition of the polishing solution, and the surface quality condition was uniform and smooth. This study introduces a novel polishing fluid for the UACMP process of silicon carbide and offers innovative insights for its application. The findings hold significant implications for the ultra-precision polishing of optical ceramics, with particular relevance to silicon carbide. In the future study, we will use the polishing liquid studied in this study to study the influence of different ultrasonic frequencies on chemical-mechanical polishing, and further study its deep mechanism, so as to improve the process of ultrasonic assisted chemical-mechanical polishing.

## Data Availability

Data is included in the article: For further questions, please contact the corresponding author.
